# Apolipoprotein E Polymorphism and Oxidative Stress in Human Peripheral Blood Cells: Can Physical Activity Reactivate the Proteasome System through Epigenetic Mechanisms?

**DOI:** 10.1155/2021/8869849

**Published:** 2021-01-02

**Authors:** Rebecca Piccarducci, Simona Daniele, Beatrice Polini, Sara Carpi, Lucia Chico, Jonathan Fusi, Filippo Baldacci, Gabriele Siciliano, Ubaldo Bonuccelli, Paola Nieri, Claudia Martini, Ferdinando Franzoni

**Affiliations:** ^1^Department of Pharmacy, University of Pisa, Via Bonanno 6, 56126 Pisa, Italy; ^2^NEST, Istituto di Nanoscienze, Consiglio Nazionale delle Ricerche, Scuola Normale Superiore, Piazza San Silvestro 12, 56127 Pisa, Italy; ^3^Department of Clinical and Experimental Medicine, University of Pisa, Via Roma 67, 56126 Pisa, Italy

## Abstract

Alzheimer's disease (AD) is characterized by proteasome activity impairment, oxidative stress, and epigenetic changes, resulting in *β*-amyloid (A*β*) production/degradation imbalance. Apolipoprotein E (ApoE) is implicated in A*β* clearance, and particularly, the ApoE *ε*4 isoform predisposes to AD development. Regular physical activity is known to reduce AD progression. However, the impact of ApoE polymorphism and physical exercise on A*β* production and proteasome system activity has never been investigated in human peripheral blood cells, particularly in erythrocytes, an emerging peripheral model used to study biochemical alteration. Therefore, the influence of ApoE polymorphism on the antioxidant defences, amyloid accumulation, and proteasome activity was here evaluated in human peripheral blood cells depending on physical activity, to assess putative peripheral biomarkers for AD and candidate targets that could be modulated by lifestyle. Healthy subjects were enrolled and classified based on the ApoE polymorphism (by the restriction fragment length polymorphism technique) and physical activity level (Borg scale) and grouped into ApoE *ε*4/non-*ε*4 carriers and active/non-active subjects. The plasma antioxidant capability (AOC), the erythrocyte A*β* production/accumulation, and the nuclear factor erythroid 2-related factor 2 (Nrf2) mediated proteasome functionality were evaluated in all groups by the chromatographic and immunoenzymatic assay, respectively. Moreover, epigenetic mechanisms were investigated considering the expression of histone deacetylase 6, employing a competitive ELISA, and the modulation of two key miRNAs (miR-153-3p and miR-195-5p), through the miRNeasy Serum/Plasma Mini Kit. ApoE *ε*4 subjects showed a reduction in plasma AOC and an increase in the Nrf2 blocker, miR-153-3p, contributing to an enhancement of the erythrocyte concentration of A*β*. Physical exercise increased plasma AOC and reduced the amount of A*β* and its precursor, involving a reduced miR-153-3p expression and a miR-195-5p enhancement. Our data highlight the impact of the ApoE genotype on the amyloidogenic pathway and the proteasome system, suggesting the positive impact of physical exercise, also through epigenetic mechanisms.

## 1. Introduction

Apolipoprotein E (ApoE) belongs to the family of apolipoproteins, i.e., proteins involved in the lipoprotein assembly, lipid transport, and metabolism by mediating interactions with receptors, enzymes, and lipid transport proteins [1]. The ApoE is mainly associated with chylomicron, very low-density lipoprotein (VLDL), and high-density lipoprotein (HDL) [2], delivering lipids through blood, cerebrospinal fluid, and lymph [3]. It is chiefly synthesized in the peripheral system by the liver and macrophages and in the central nervous system (CNS) by astrocytes, by microglia, or in pathological conditions by neurons [4]. The ApoE is a 34 kDa glycoprotein of 299 amino acids, encoded by the ApoE gene located on chromosome 19q13.2, which exists in three different isoforms: ApoE *ε*2, ApoE *ε*3, and ApoE *ε*4. Each isoform differs from the others for the presence of a cysteine (C) or arginine (R) in positions 112 and 158 of the amino acid sequence of the protein. These dissimilarities in the primary sequence lead to alterations in the structure and function of the ApoE isoforms [5].

Beyond the function in the regulation of the transport and metabolism of lipids, ApoE is implicated in the maintenance of normal brain functions, playing an important role in neurological disorders (NDs) [6]. In the CNS, ApoE regulates the clearance of amyloid beta (A*β*), which is a common hallmark of NDs, mainly associated with Alzheimer's disease (AD). Notably, ApoE *ε*4 is the main genetic risk factor that leads to AD, especially when the genotype is homozygous (*ε*4/*ε*4) [4]. Interestingly, the *ε*4 isoform of ApoE shows a reduced affinity to A*β* compared to the other isoforms, decreasing A*β* clearance and giving rise to the formation of A*β* toxic oligomers, which constitute the extracellular plaques, a typical pathogenic feature of AD [6].

Many studies have extensively demonstrated the association between the ApoE genotype and oxidative stress. Particularly, the *ε*4 is the lowest effective isoform of ApoE in protecting cells from oxidative stress, both *in vitro* and *in vivo* [7]. Oxidative stress is the main process that initiates and enhances the pathological processes that characterized NDs. Of note, a significant degree of oxidative damage is associated with the accumulation of A*β* in the brain of AD patients [8, 9]. Indeed, besides the imbalance between the dangerous generation of reactive oxygen species (ROS) and the lacking ability of the biological system to remove them, AD is characterized by the incapacity to maintain a homeostatic balance between amyloid production and its degradation, resulting in the direct inhibition of proteasome activity and indirect elevation of oxidative stress, both of which contribute to protein dysfunction [10]. In this sense, accumulating evidence indicates that the dysfunction of the ubiquitin-proteasome system (UPS) is a key factor to initiate and aggravate the pathogenesis of NDs. On the other hand, A*β* accumulation has been proven to reduce proteasomal activity in cultured neurons [11]. From a molecular point of view, misfolded protein accumulation and aggregation induce an atypical production of ROS that modifies the ubiquitin E3-ligase Kelch-like ECH-associated protein 1 (Keap1), leading to the release, stabilization, and nuclear localization of the nuclear factor erythroid 2-related factor 2 (Nrf2), finally increasing the transcription of antioxidant response element (ARE) genes [12]. Physiologically, Nrf2 upregulates the proteasome system subunits, protecting cells from the accumulation of toxic proteins [12, 13]. Moreover, Nrf2 has been demonstrated to be a negative regulator of *β*-secretase 1 (BACE1) expression, thus ameliorating A*β* pathology and cognitive deficits [14]. However, altered levels of Nrf2 and of the ubiquitinated form have been found in NDs, including AD [12, 15]. Furthermore, decreased levels of Nrf2-dependent target gene expression have been found in ApoE *ε*4 compared to other isoforms [16], strengthening the link between ApoE polymorphism, proteasome activity, and neurodegenerative processes.

In addition to the link with genetics, oxidative stress and proteasome activity impairment are closely related to epigenetic processes [17]. Epigenetics includes several biological processes involved in the regulation of gene expression without interfering with the DNA sequence itself [18].

Among epigenetic modulators, growing evidence underlines the pivotal role of histone acetylation and deacetylation on gene expression. Furthermore, also microRNAs (miRNAs), small noncoding RNAs, are known for their ability to strongly regulate gene expression.

The link between miRNA and oxidative stress is bidirectional: oxidative stress induces the up- or downregulation of several miRNAs and many miRNAs can regulate oxidative stress response [19, 20]. In addition to the strong involvement in oxidative stress, miRNAs are able to regulate several processes responsible for AD development, such as mechanisms of proteasome impairment, i.e., accumulation of A*β* peptides and tau phosphorylation [21–23]. For instance, miR-153-3p appears to be involved in AD progression by targeting either amyloid precursor protein (APP) or Nrf2 [19, 24, 25]. Similarly, miR-193a-3p has been reported to modulate A*β* accumulation by targeting BACE1 and APP [26], and it was recently identified as an oxidative stress-responsive miRNA [27].

Besides the genetic risk factors, the environmental risk factors that lead to AD are numberless, such as mainly the diet and physical activity [28, 29].

Globally, it has been demonstrated that the Mediterranean diet is associated with a reduced risk to AD [30, 31], while a deficiency of vitamin D and selenium has been suggested to be predisposing factors for AD [32–34]. Interestingly, ApoE polymorphism has been demonstrated to strictly correlate with the concentration of vitamin D or selenium [33, 35].

Moreover, many studies have demonstrated that regular and moderate physical activity can prevent or reduce the progression of NDs [36]. Physical exercise has demonstrated to upregulate the antioxidant capability, modulate oxidative stress, and increase degradation of amyloidogenic oligomers [36]. Furthermore, aerobic exercise has been shown to upregulate UPS in healthy mice [37]. Also, all epigenetic agents are strictly influenced by environmental and lifestyle factors, including physical exercise [38–41].

In this regard, we have recently demonstrated that physical activity modulates the accumulation of ND-related misfolded proteins in peripheral cells, both in healthy subjects and in ND patients, and plays a pivotal role in maintaining the physiological erythrocyte well-being and plasma antioxidant capability in healthy volunteers [42–45].

Notably, erythrocytes are particularly susceptible to oxidative stress, due to their high vulnerability to peroxidation and, consequently, to the accumulation of misfolded proteins, such as A*β* [46–48]. Particularly, the exposure of this kind of peripheral cells to oxidative conditions, such as exercise and hypoxia, leads to lipid peroxidation, cellular morphology changes, and protein alterations [48], thus considering erythrocytes a good peripheral model to study biochemical alterations related to NDs, involving protein misfolding accumulation, antioxidant defences, and proteasome activity.

Therefore, the present study was aimed at investigating the influence of the ApoE polymorphism and the physical activity on the oxidative stress levels, the amyloidogenic pathway of A*β* production/accumulation, and the Keap1-Nrf2-mediated proteasome functionality. Finally, the epigenetic mechanisms around the amyloidogenic pathway and Keap1-Nrf2 axis were evaluated considering the expression of histone deacetylase 6 (HDAC6) and the modulation exerted by two key miRNAs (miR-153-3p and miR-195-5p). All these aspects were evaluated in peripheral cells, i.e., erythrocytes, and plasma of healthy subjects classified on the basis of ApoE polymorphism and the level of physical activity [49].

## 2. Materials and Methods

### 2.1. Recruitment of Healthy Volunteers and Genotyping of ApoE Polymorphism

The healthy subjects (forty-two age- and sex-matched Table 1) were recruited from the Sports Medicine Unit (Department of Clinical and Experimental Medicine, University of Pisa). This study was approved by the Ethics Committee of the Great North West Area of Tuscany (271/2014 to F.F.), and it was carried out in accordance with the Declaration of Helsinki. All subjects gave informed consent to participate in the study. Fully informed consent was obtained from each subject entering the study [45].

The blood was collected from each subject, and subsequently, genomic DNA was extracted from the whole blood. The restriction fragment length polymorphism (RFLP) technique has been employed to classify the subjects into ApoE *ε*4 carriers and non-*ε*4 carriers. Briefly, the polymerase chain reaction (PCR) was made with 1.5 pmol of each primer (forward 5′-TCG-GCCGCA-GGG-CGC-TGA-TGG-3′ and reverse 5′-CTCGCG-GGC-CCC-GGC-CTG-GTA-3′), 250 *μ*mol/L dNTPs, GC-rich (10% of the final volume), 2 units of Taq DNA polymerase (Applied Biosystems Inc., Branchburg, NJ), 10 ng/*μ*L of genomic DNA, 25 mM MgCl_2_, and buffer 10X. A thermal cycler (PerkinElmer) was employed for reactions: one cycle at 94°C for 6 min, 30 cycles at 94°C for 40 s, 67°C for 30 s, 72°C for 45 s, and a final extension at 72°C for 5 min. The amplified fragments, resulting from digestion with 3 U of HhaI restriction enzyme, were divided through agarose (5%) gel electrophoresis. The restriction patterns were displayed using ethidium bromide staining and UV light.

The subjects' genotypes were established by using the ABI PRISM310 Automated Sequencer (Applied Biosystems, Foster City, CA, USA). Thus, the subjects have been classified into ApoE *ε*4 carriers (sixteen, age- and sex-matched, Table 1), who included heterozygote subjects *ε*4/*ε*3, and ApoE non-*ε*4 carriers (twenty-six, age- and sex-matched, Table 1), who included heterozygote subjects (*ε*2/*ε*3) or homozygote ones (*ε*3/*ε*3). The lowered number of ApoE *ε*4 carriers is due to a reduced extent of this genotype in the human race compared to other polymorphisms of the same protein (ApoE *ε*2 or ApoE *ε*3) [5, 42]. When the DNA concentration was too low to allow correct discrimination of ApoE alleles by RFLP, subjects' genotypes were established by using the ABI PRISM310 Automated Sequencer [49].

The subjects were grouped into ApoE *ε*4 carriers and ApoE non-*ε*4 carriers, on the basis of ApoE polymorphism (Section 2.1). In each group, the subjects are further classified into non-active (NA) and active (A), on the basis of the physical activity level (Section 2.3). The number of recruited subjects (*N*), age (years), and sex (M/F) are indicated. Values are expressed as mean ± SD.

### 2.2. Clinical Parameters of the Enrolled Subjects

Italian healthy subjects with an upper-middle socioeconomic status and a Mediterranean diet have been recruited for the current study. Each participant shows neither cardiovascular disease nor other major medical disorders, which were thus established by clinical history, physical examination, blood pressure, blood chemistry, haematology, urine analysis, and basal and stress electrocardiography, with a maximal graded cycle ergometry test executed by a cardiologist blinded to the other data [42, 43, 50]. Familial AD cases were excluded from subject sampling.

Generally, the major inclusion criteria were as follows: diastolic arterial blood pressure lower than 90 mmHg, systolic arterial blood pressure lower than 140 mmHg, body mass index lower than 30 kg/m^2^, plasma triglycerides from 30 to 150 mg/mL, total plasma cholesterol ranging from 120 to 220 mg/mL, and HDL cholesterol from 26 to 75 mg/mL. Smokers and subjects in treatment with drug/nutraceutical were excluded from the study [42, 43, 49].

### 2.3. Levels of Physical Activity of the Enrolled Subjects

The participants were grouped into non-active (NA) and active (A) based on the habit questionnaire (Table 1). All the recruited subjects classified as active practiced moderate aerobic fitness, mainly running. According to the World Health Organization (WHO) [51], a non-active subject performs less than 150 minutes per week of physical activity. Moreover, the Borg Rating and Perceived Exertion (RPE) scale has been employed to evaluate the intensity level of physical activity [52]. The scale ranges from 6 to 20: 6 corresponds to no exertion at all, 7.5 to extremely light, 9 to very light, 11 to light, 13 to somewhat hard, 15 to hard, 17 to very hard, 19 to extremely hard, and 20 to maximal exertion [42, 49].

### 2.4. Blood Specimen Collection

The whole blood was collected from each volunteer at least 48 h later the last exercise bout, and it was stored into an anticoagulant EDTA tube. The blood was centrifuged at 200 × *g* at 4°C for 10 min to separate erythrocytes from plasma.

The plasma supernatant was isolated and stored at -20°C until use. The erythrocyte pellet was suspended in 3 mL of PBS, centrifuged at 1000 × *g* for 10 min, and washed with PBS. Following further centrifugation at 1500 × *g* for 10 min, the isolated erythrocytes were stored at -20°C until use [42, 49].

### 2.5. Assessment of the Total Antioxidant Capability (AOC) in Plasma

The total antioxidant capability (AOC) in plasma was assessed using the total oxyradical scavenging capacity (TOSC) assay, a gas chromatographic assay able to define the oxyradical scavenging capacity of biological fluids [42, 43, 53]. Hydroxyl radicals were generated at 35°C by the iron plus ascorbate-driven Fenton reaction (1.8 mM Fe^3+^, 3.6 mM EDTA, and 180 mM ascorbic acid in 100 mM PBS, pH 7.4). Reactions with 0.2 mM KMBA (alpha-keto gamma-methylthiobutyric acid) were performed in 10 mL vials sealed with gas-tight Mininert valves (Supelco, Bellefonte, PA) in a final volume of 1 mL. Ethylene production was quantified by gas chromatographic analysis of 200 *μ*L aliquots taken from the headspace of vials at timed intervals during the reaction (Hewlett-Packard gas chromatograph, HP 7820A Series, Andoven, M, equipped with a Supelco DB-1 capillary column and a flame ionization detector (FID)). Total ethylene formation was measured from the area under the curves that best define the experimental points obtained for control reactions and after the addition of plasma during the reaction [42, 53, 54]. The equation TOSC = 100 − (SA/CA × 100) was used to determine the TOSC values: SA is the area under the curve (AUC) for the sample and CA is the control reaction. A TOSC value of 100 is correlated with a sample able to suppress the ethylene formation, while a negative TOSC value is attributed to a prooxidant sample. A TOSC value of 0 corresponds to a sample without scavenging capacity [55]. Each experiment was performed twice to consider the intrinsic variability of the method. The results were indicated in TOSC units/mL [42, 49, 53, 56].

### 2.6. Quantification of Amyloid Beta (A*β*) in Erythrocytes

The concentration of A*β* in erythrocytes was measured by an enzyme-linked immunosorbent assay (ELISA), as described [42, 43]. The plate was precoated with a specific antibody to A*β* (sc-9129, Santa Cruz Biotechnology), diluted in poly-L-ornithine, and maintained overnight at 4°C. Following washing with PBS-T (PBS, containing 0.01% Tween 20), to block nonspecific sites, BSA 1% was added and incubated for 2 h at 37°C. After washes with PBS-T, erythrocytes (0.05 mg/100 *μ*L) were added to each well (100 *μ*L/well) and incubated for 1 h at 25°C. Then, a polyclonal antibody to A*β* (sc-5399, Santa Cruz Biotechnology) was employed and incubated for 1.5 h at 25°C. Consequently, an HRP antibody (Santa Cruz Biotechnology) was added to each well and incubated for 1 h at 37°C. The 3,3′,5,5′-tetramethylbenzidine (TMB) (Thermo Scientific) and, consequently, the stop solution (H_2_SO_4_) were added, and the absorbance was read at 450 nm (EnSight Multimode Plate Reader, PerkinElmer). All measurements were performed in duplicate. The standard curve for ELISA was constructed using recombinant human A*β* solution at different concentrations [42, 43, 45, 49].

### 2.7. Evaluation of Erythrocyte Amyloid Precursor Protein (APP)

The amyloid precursor protein (APP) levels in erythrocytes were evaluated through a sandwich ELISA kit (Human Amyloid Precursor Protein, ELISA kit, MyBioSource, #MBS731247).

Erythrocytes (50 *μ*L), isolated from the whole blood as already described (Section 2.4), were diluted to 1 : 10 in PBS (pH = 7.0-7.2) and incubated in the wells of the precoated plate, together with a balance solution (5 *μ*L) and conjugate (100 *μ*L), for 1 h at 37°C. Then, each well was thoroughly washed to remove all unbound components. Substrate solutions were added to each well. After a short incubation period necessary for the substrate to react with the enzyme (HRP), and following the addition of sulphuric acid to terminate the enzyme-substrate reaction, the absorbance was read at 450 nm (EnSight Multimode Plate Reader, PerkinElmer). A standard curve was designed relating the intensity of the colour (O.D.) to the concentration of standards. The APP concentration (ng/mg of total proteins) was interpolated from the standard curve [49].

### 2.8. Evaluation of Erythrocyte Expression of *β*-Secretase 1 (BACE1)

The *β*-secretase 1 (BACE1) amount in erythrocytes was evaluated through a sandwich ELISA kit (human beta-secretase 1, ELISA kit, Thermo Scientific Pierce, #EHBACE1).

Erythrocytes (100 *μ*L), isolated from the whole blood as already described (Section 2.4), were diluted to 1 : 25 in 1X assay diluent and incubated for 2.5 h at room temperature with gentle shaking. Following washing, a 1X biotinylated antibody (100 *μ*L) was added to each well and incubated for 1 h at room temperature with gentle shaking. After washing, streptavidin-HRP solution (100 *μ*L) was added to each well and incubated for 45 min at room temperature with gentle shaking. Then, the wells were washed and TMB substrate (100 *μ*L) was added 5to each well. The colorimetric reaction was stopped by the addition of the stop solution (50 *μ*L) to each well. The absorbance was read at 450 nm (EnSight Multimode Plate Reader, PerkinElmer). The standard curve was generated by plotting the absorbance obtained from each standard. The BACE1 concentration (ng/mL) was quantified according to the standard curve [49].

### 2.9. Quantification of the Total Amount of Erythrocyte Nuclear Factor Erythroid 2-Related Factor 2 (Nrf2)

The nuclear factor erythroid 2-related factor 2 (Nrf2) was quantified in erythrocytes by a high-throughput assay that combines quick ELISA with a sensitive and specific nonradioactive one for transcription factor activation (Nrf2 Transcription Factor Assay Kit, colorimetric, Abcam, #ab207223). Through this assay, only active Nrf2 that is present in the sample is detected by a primary antibody that recognizes an epitope of Nrf2 accessible only when the protein is activated.

Erythrocytes (10 *μ*L, i.e., 5-20 *μ*g), separated from the whole blood as described above (Section 2.4), were diluted in the completed binding buffer and incubated for 1 h at room temperature with mild agitation (100 rpm). After extensive washes, a primary antibody (100 *μ*L) was added and incubated for 1 h at room temperature without shaking. Following washing, a secondary antibody (100 *μ*L) was added and incubated for 1 h at room temperature without shaking. Then, the wells were washed and the developing solution was added and incubated. After the addition of the stop solution, the absorbance was read at 450 nm (EnSight Multimode Plate Reader, PerkinElmer). The Nrf2 amount was calculated from Nrf2 activation absorbance and normalized to the absorbance of the total proteins in the sample (*μ*g/*μ*L) [49].

### 2.10. Measurement of Erythrocyte Histone Deacetylase 6 (HDAC6)

Histone deacetylase 6 (HDAC6) was detected in erythrocytes with a competitive ELISA kit (Human Histone Deacetylase 6 (HDAC6) Elisa Kit, Competitive ELISA, MyBioSource, #MBS7254230).

Erythrocytes (100 *μ*L), isolated from the whole blood as already described (Section 2.4), were diluted to 1 : 10 in PBS (pH = 7.0-7.2) and incubated in the wells of the precoated plate, together with a balance solution (10 *μ*L) and conjugate (50 *μ*L), for 1 h at 37°C. Afterward, the wells were carefully washed to remove all unbound components. Substrate solutions were added to each well and incubated for a few minutes. Following the addition of sulphuric acid to terminate the enzyme-substrate reaction, the absorbance was read at 450 nm (EnSight Multimode Plate Reader, PerkinElmer). A standard curve was designed relating the intensity of the colour (O.D.) to the concentration of standards. The HDAC6 concentration (pg/mg of total proteins) was interpolated from the standard curve [49].

### 2.11. Expression of Plasma Kelch-Like ECH-Associated Protein 1 (Keap1)

The expression of plasma Kelch-like ECH-associated protein 1 (Keap1) was detected by western blot analysis.

Briefly, plasma (10 *μ*g of total proteins, quantified through the Lowry assay), opportunely isolated from the whole blood as described above (Section 2.4), with additional Laemmli solution, was resolved by electrophoresis using a 4-20% Criterion TGX stain-free precast gel (Bio-Rad, #5678094). Afterward, the samples were transferred by using the Trans-Blot Turbo Transfer System (Bio-Rad) to Trans-Blot Turbo Midi 0.2 *μ*M PVDF membrane (Bio-Rad, #1704157). Then, the membrane was incubated for at least 1 h with a buffer able to block nonspecific sites (5% milk). A primary antibody against Keap1 (rabbit, #AV38981, Sigma-Aldrich) was used and incubated overnight at 4°C, under continuous agitation. Following the incubation with a secondary HRP-conjugated antibody, protein bands were detected with a chemiluminescent substrate (Clarity Western ECL Substrate, Bio-Rad, #1705061). Densitometry was performed by ImageJ software. Images were obtained in different western blots using a reference standard for each running gel, due to the impossibility to show all the samples at the same time [49].

### 2.12. Analysis of the Expression of Circulating miRNAs

Plasma, isolated from the whole blood as previously described (Section 2.4), was processed by using the miRNeasy Serum/Plasma Mini Kit (Qiagen, Hilden, Germany) to isolate total RNA, including microRNAs (miRNAs). Retrotranscription was carried out using the miRCURY LNA miRNA RT Kit (Qiagen, Hilden, Germany), and the obtained cDNA was diluted to 1 : 30, immediately before use. Real-time PCR was run on the MiniOpticon CFX 48 Real-Time PCR Detection System (Bio-Rad, Hercules, CA, USA) using the miRCURY LNA miRNA SYBR Green PCR and specific miRCURY LNA miRNA PCR Assay (Qiagen, Hilden, Germany), as previously reported [57]. The miRCURY Primer Assay specific for hsa-miR-195-5p (MIMAT0000461), hsa-miR-153-3p (MIMAT0000439), and hsa-miR-93-5p (MIMAT0000093) was purchased from Qiagen (Hilden, Germany).

The relative miRNA expression was calculated using the Ct method and normalized on miR-93-5p. Several pieces of evidence reported high stability of miR-93-5p in biofluids [58–61]; thus, miR-93-5p was suggested as a plasmatic reference gene in the manufacturer's handbook. According to this, the expression levels of miR-93-5p in plasma samples of our cohort showed comparable expression levels without significant difference among groups (data not shown) [49].

### 2.13. Statistical Analysis

The data are shown as the mean value ± SD (standard deviation). The data relative to A*β*, APP, and BACE1 were depicted as median values. The sample size calculator was used to estimate the accuracy of the results. Accordingly, the study group required 40 patients to obtain the same difference with *α* = 0.05 and statistical power of 85%.

Kolmogorov–Smirnov tests were used for data meeting the assumption of a normality distribution. The variance between groups was statistically significant using Bartlett's test. One-way and two-way analysis of variance (ANOVA) tests were used to evaluate differences among groups for data meeting the assumption of homogeneity of variance. Pearson correlation analysis and *t*-tests were applied when only two groups were present for data with distributions that met parametric assumptions. Chi-square tests (Pearson's, Yates-adjusted, or Fisher's exact test according to sample size), Mann–Whitney *U* tests, and Spearman correlation analysis were employed in situations where parametric assumptions were not met. Tukey's multiple comparison test was applied for the densitometry analysis. Statistical analysis for miRNA expression was performed using the Kruskal-Wallis (nonparametric) followed by Dunn's multiple comparison test.

Correlation between variables was determined by linear regression analysis, while interactions between variables were analysed by correlation and multiple regression analyses. *P* values < 0.05 were considered significantly different. All statistical analyses were carried out by using commercial software (GraphPad Prism, version 7.0; GraphPad Software Inc., San Diego, CA) [42, 45, 49, 54].

## 3. Results

### 3.1. Descriptive Statistics

The whole cohort of healthy volunteers (8) was divided into ApoE *ε*4 carriers (mean age 39 ± 14) and ApoE non-*ε*4 carriers (mean age 40 ± 13), and based on the results of the Borg score (Section 2.3), the subjects were additionally classified into non-active (NA) and active (A).

The population did not show significant differences in age, sex distribution, and body mass index (BMI) [49].

### 3.2. Plasma Antioxidant Capability (AOC)

The antioxidant capability (AOC) was evaluated in the plasma of the whole cohort.

**Figure 1:**
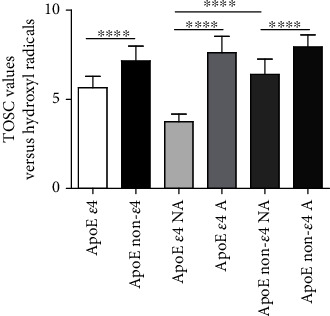
Plasma AOC levels detected by the TOSC assay. The plasma AOC towards hydroxyl radicals was detected by the TOSC assay, as described in Materials and Methods: high TOSC values are associated with elevated antioxidant capacity. The data are shown as the mean value ± SD and are representative of three independent experiments (*n* = 3). *P* values were adjusted with the unpaired *t*-test: ^∗∗∗∗^*P* < 0.0001 between the indicated subgroups. GraphPad Prism 7 was used to create the figure.

The AOC levels versus hydroxyl radicals (Figure 1) were significantly decreased in ApoE *ε*4 compared to non-*ε*4 carriers (*P* < 0.0001), underling the impact of ApoE polymorphism on the AOC. A significantly higher AOC was evidenced in active compared to non-active subjects, in both ApoE *ε*4 (*P* < 0.0001) and non-*ε*4 carrier groups (*P* < 0.0001). Moreover, among non-active subjects, the non-*ε*4 carriers presented increased plasma AOC levels compared to *ε*4 ones (*P* < 0.0001), while no differences have been identified among active subjects (*P* = 0.3649) [49].

### 3.3. Erythrocyte Amyloid Beta (A*β*)

The concentration of A*β* was evaluated in the erythrocytes of the whole cohort.

**Figure 2:**
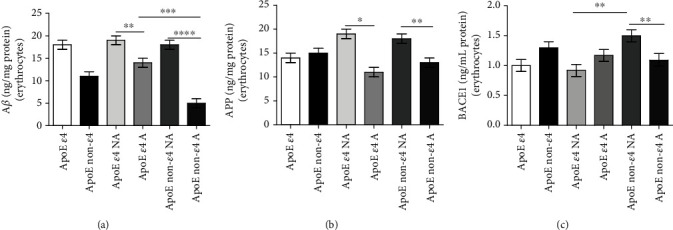
Erythrocyte A*β*, APP, and BACE1 accumulation measured by using a sandwich ELISA kit. The whole blood was collected by volunteers, and erythrocytes were isolated through sequential centrifugations, as described in Materials and Methods. A sandwich ELISA kit was employed to measure the erythrocyte concentration of A*β* (a), APP (b), and BACE1 (c). The data are shown as the median value with range and are representative of three independent experiments (*n* = 3). *P* values were adjusted with the unpaired *t*-test: ^∗^*P* < 0.05, ^∗∗^*P* < 0.01, ^∗∗∗^*P* < 0.001, and ^∗∗∗∗^*P* < 0.0001 between the indicated subgroups. GraphPad Prism 7 was used to create the figure.

The amount of A*β* (Figure 2(a)) showed a trend of increase in ApoE *ε*4 carriers compared to non-*ε*4 ones (*P* = 0.4261). Moreover, in both *ε*4 and non-*ε*4 carrier groups, non-active subjects showed higher A*β* levels than active subjects (*ε*4: *P* = 0.0060; non-*ε*4: *P* < 0.0001).

Furthermore, among active subjects, *ε*4 carriers displayed an increased amount of A*β* in erythrocytes compared to non-*ε*4 carriers (*P* = 0.0004), while no differences have been identified in non-active groups (*P* = 0.8003) [49].

### 3.4. Erythrocyte Amyloid Precursor Protein (APP)

The amyloid precursor protein (APP) concentration was assessed in the erythrocytes of the recruited subjects.

The total amount of APP (Figure 2(b)) was comparable in ApoE *ε*4 and non-*ε*4 carriers (*P* = 0.8370), underlining that the APP levels in erythrocytes are independent of ApoE polymorphism. Generally, non-active subjects showed increased erythrocyte APP levels when compared to the active ones (*ε*4 carriers: *P* = 0.0119; non-*ε*4: *P* = 0.0018) [49].

### 3.5. Erythrocyte Expression of *β*-Secretase 1 (BACE1)

The expression of *β*-secretase 1 (BACE1) in erythrocytes was detected among the recruited subjects.

A trend of increase in BACE1 levels in erythrocytes (Figure 2(c)) has been identified in ApoE non-*ε*4 carriers compared with the *ε*4 carrier group (*P* = 0.1681). Moreover, among non-*ε*4 carriers, non-active subjects showed an increased BACE1 amount compared to the active ones (*P* = 0.0067), while no differences have been identified among *ε*4 carriers (*P* = 0.2467).

Furthermore, among non-active subjects, non-*ε*4 carriers presented increased BACE1 levels compared to *ε*4 ones (*P* = 0.0015) [49].

### 3.6. Erythrocyte Nuclear Factor Erythroid 52-Related Factor 2 (Nrf2)

**Figure 3:**
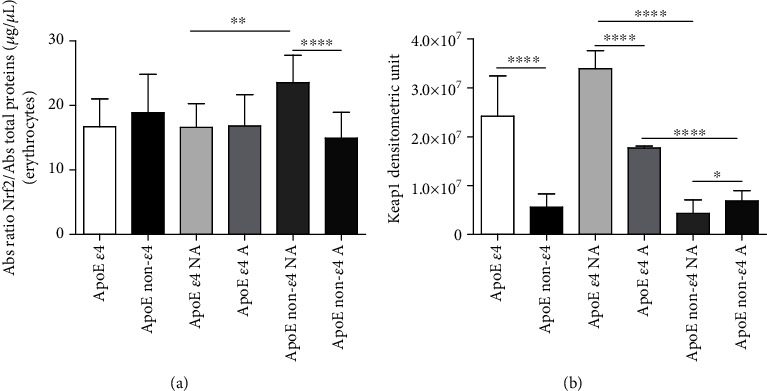
Erythrocyte Nrf2 and plasma Keap1 quantified by the transcription factor assay and western blot analysis, respectively. (a) Nrf2 was measured in erythrocytes, isolated from the whole blood of healthy volunteers, through a high-throughput assay that combines quick ELISA with a sensitive and specific nonradioactive one for transcription factor activation. By this kind of assay, only the activated Nrf2 is detected (Materials and Methods). (b) Plasma collected from healthy volunteers was considered to identify the presence of the Keap1 through western blot analysis that is shown as the densitometric unit of the bands (Materials and Methods). The data are shown as the mean value ± SD and are representative of three independent experiments (*n* = 3). *P* values were adjusted with the unpaired *t*-test: ^∗^*P* < 0.05, ^∗∗^*P* < 0.01, and ^∗∗∗∗^*P* < 0.0001 between the indicated subgroups. GraphPad Prism 7 was used to create the figure.

The nuclear factor erythroid 2-related factor 2 (Nrf2) accumulation was evaluated in erythrocytes of the recruited subjects. The detection of Nrf2 in the erythrocytes (Figure 3(a)) of ApoE *ε*4 carriers versus non-*ε*4 carriers did not reveal significant differences (*P* = 0.2128), even if among non-active subjects, non-*ε*4 carriers displayed an increased amount of Nrf2 compared to *ε*4 carriers (*P* = 0.0011). Of note, in the non-*ε*4 subgroup, the data showed a higher Nrf2 amount in non-active than in active subjects (*P* < 0.0001) [49].

### 3.7. Plasma Kelch-Like ECH-Associated Protein 1 (Keap1)

Kelch-like ECH-associated protein 1 (Keap1) was assessed in the plasma of the recruited subjects.

Keap1 concentrations (Figure 3(b)) were significantly lower in non-*ε*4 carriers compared to *ε*4 carriers, in the whole group (*P* < 0.0001) and among both non-active (*P* < 0.0001) and active (*P* < 0.0001) subjects. Moreover, among ApoE *ε*4 carriers and non-*ε*4 carriers, Keap1 was significantly lower in active subjects compared to non-active ones (*ε*4: *P* < 0.0001; non-*ε*4: *P* = 0.0138) [49].

### 3.8. Erythrocyte Histone Deacetylase 6 (HDAC6)

The evaluation of the histone deacetylase 6 (HDAC6) concentration was performed in the erythrocytes of recruited subjects.

**Figure 4:**
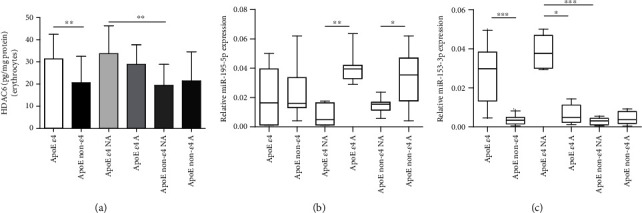
Erythrocyte HDAC6 concentration quantified by using a competitive ELISA kit and circulating expression of miR-195-5p and miR-153-3p. (a) HDAC6 was quantified in erythrocytes, isolated from the whole blood of the total cohort, employing a competitive ELISA kit (Materials and Methods). The data are shown as the mean value ± SD and are representative of three independent experiments (*n* = 3). *P* values were adjusted with the unpaired *t*-test: ^∗∗^*P* < 0.01 between the indicated subgroups. The expression of miR-195-5p (b) and miR-153-3p (c) was analysed in plasma samples of non-active and active subjects, classified based on ApoE *ε*4 polymorphism. The analysis was performed through the miRNeasy Serum/Plasma Mini Kit, as described in Materials and Methods. The relative expression was calculated by the Ct method and normalized on miR-93-5p. Statistical analysis was performed by Kruskal-Wallis (nonparametric) followed by Dunn's multiple comparison test. ^∗^*P* < 0.05, ^∗∗^*P* < 0.01, and ^∗∗∗^*P* < 0.001 between the indicated subgroups. GraphPad Prism 7 was used to create the figure.

Concerning this evidence, the total erythrocyte amount of HDAC6 (Figure 4(a)) was measured. ApoE non-*ε*4 carriers showed lower levels of HDAC6 in erythrocytes than ApoE *ε*4 carriers in the whole cohort (*P* = 0.0062) and in the non-active subgroup (*P* = 0.0083), while among active subjects, it was comparable (*P* = 0.1750). Beyond the emerged differences on the basis of ApoE polymorphism in the whole cohort, the concentration of HDAC6 in erythrocytes was comparable between active and non-active subjects (*ε*4 carriers, *P* = 0.3918; non-*ε*4 carriers, *P* = 0.6574) [49].

### 3.9. Expression Levels of Circulating miRNAs

The levels of circulating miRNAs were assessed in the plasma of the whole cohort.

The levels of miR-195-5p (Figure 4(b)) were almost comparable among ApoE *ε*4 and non-*ε*4 carriers. Nevertheless, a significant difference inside these groups appeared when active and non-active subjects were separately analysed. In particular, the circulating miR-195-5p levels were significantly higher in active than in non-active subjects, with a greater difference observed between the groups when the *ε*4 polymorphism was present (8.2-fold change (*P* = 0.0013) for ApoE *ε*4 and 2.3-fold (*P* = 0.0489) for ApoE non-*ε*4).

As regards the expression of miR-153-3p (Figure 4(c)), it was significantly lower in ApoE non-*ε*4 carriers than in *ε*4 ones (*P* = 0.0002). Furthermore, in *ε*4 carriers, the expression of miR-153-3p was significantly higher (6.1-fold change, *P* = 0.045) in plasma of non-active compared to active subjects. Increased levels (12-fold, *P* = 0.0003) appeared also from the comparison between non-active subjects belonging to the different polymorphic states. Instead, no significant differences were detected among active subjects from the *ε*4 and non-*ε*4 groups, respectively [49].

### 3.10. Correlation of Plasma and Erythrocyte Parameters with the Plasma Antioxidant Capability (AOC)

No significant correlation with age was evidenced for all the analysed parameters.

**Figure 5:**
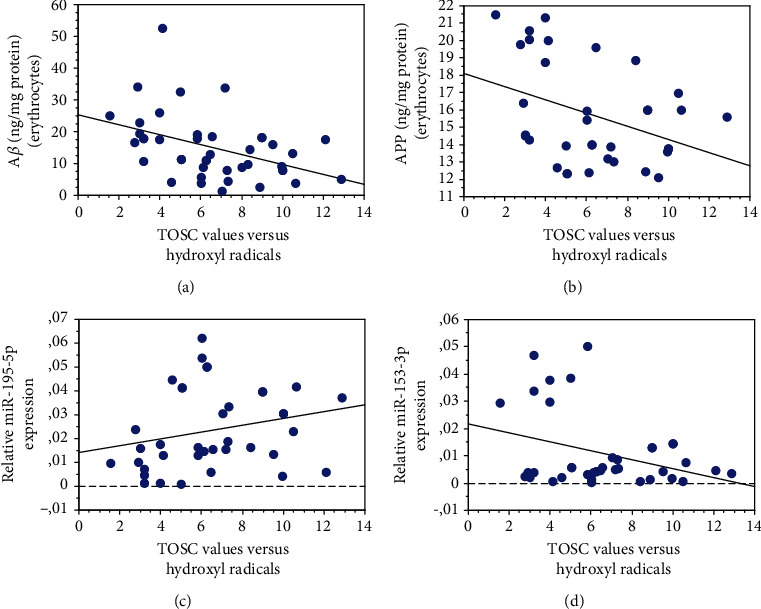
Correlation of erythrocyte and plasma parameters with AOC expressed as TOSC values versus hydroxyl radicals. Correlation analysis between A*β* (a), APP (b), miR-195-5p (c), and miR-153-3p (d) with plasma AOC. Correlation between variables was determined by simple linear regression analysis, using the StatView program (Abacus Concepts, Inc., SAS Institute, Cary, NC). *P* and *R*^2^ values obtained for each correlation are reported in the respective panel.

The plasma AOC showed an inverse correlation with A*β* (Figures 5(a), *P* = 0.0048, *R*^2^ = 0.183) or APP (Figure 5(b), *P* = 0.0255, *R*^2^ = 0.142) accumulation in erythrocytes.

Interestingly, a significant positive correlation was observed between plasma AOC and miR-195-5p (Figure 5(c), *P* = 0.0456, *R*^2^ = 0.158). In contrast, a negative correlation was evidenced for miR-153-3p (Figure 5(d), *P* = 0.0378, *R*^2^ = 0.111).

The plasma AOC level was not significantly related to the other examined parameters (BACE1: *P* = 0.8702; Nrf2: *P* = 0.8655; and HDAC6: *P* = 0.8660) [49].

### 3.11. Correlation of Plasma and Erythrocyte Parameters with the Level of Physical Activity

**Figure 6:**
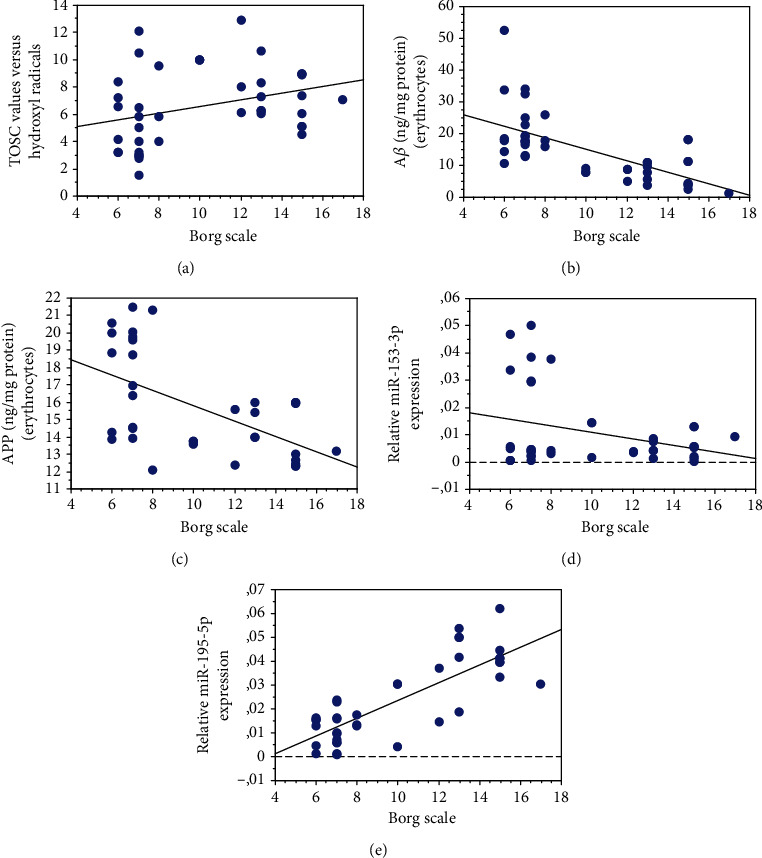
Correlation of plasma and erythrocyte parameters with the physical activity level expressed as the Borg scale. Correlation analysis between AOC (a), A*β* (b), APP (c), miR-153-3p (d), and miR-195-5p (e) with physical activity. Correlation between variables was determined by simple linear regression analysis, using the StatView program (Abacus Concepts, Inc., SAS Institute, Cary, NC). *P* and *R*^2^ values obtained for each correlation are reported in the respective panel.

The physical activity level showed a direct correlation with the plasma AOC (Figure 6(a), *P* = 0.0408, *R*^2^ = 0.109), as previously obtained in a similar cohort of subjects [42, 45].

An inverse correlation was observed between the level of physical exercise and erythrocyte accumulation of A*β* (Figure 6(b), *P* < 0.0001, *R*^2^ = 0.412) or its precursor APP (Figure 6(c), *P* = 0.04, *R*^2^ = 0.316). These data confirm that the protein levels in these blood cells may be influenced by exercise.

Interestingly, the amount of physical exercise was inversely related to the levels of miR-153-3p (Figure 6(d), *P* = 0.0464, *R*^2^ = 0.103). In contrast, a strong positive correlation was observed with miR-195-5p (Figure 6(e), *P* = 0.0078, *R*^2^ = 0.647).

The Borg score was not significantly related to the other examined parameters (BACE1: *P* = 0.1289; HDAC6: *P* = 0.5731) [49].

### 3.12. Correlation with A*β* Synthesis and Accumulation in Erythrocytes

**Figure 7:**
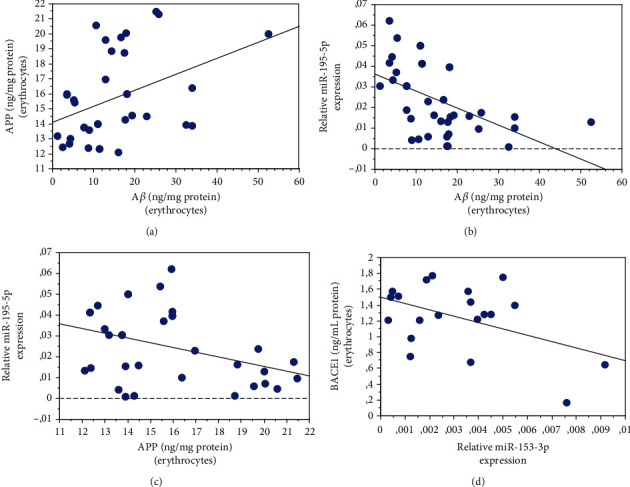
Correlation of erythrocyte and plasma parameters with erythrocyte A*β* accumulation. Correlation analysis between erythrocyte A*β* and APP (a) and plasma miR-195-5p (b). Correlation analysis between erythrocyte APP and plasma miR-195-5p (c). Correlation analysis between erythrocyte BACE1 concentrations and plasma miR-153-3p (d). Correlation between variables was determined by simple linear regression analysis, using the StatView program (Abacus Concepts, Inc., SAS Institute, Cary, NC). *P* and *R*^2^ values obtained for each correlation are reported in the respective panel.

As expected, A*β* accumulation in erythrocytes showed a positive correlation with APP (Figure 7(a), *P* = 0.0166, *R*^2^ = 0.162). In contrast, a significant inverse correlation was observed between the erythrocyte's levels of A*β* and plasma miR-195-5p (Figure 7(b), *P* = 0.0011, *R*^2^ = 0.260). The latter's negative correlation was also observed between erythrocyte APP concentration and plasma miR-195-5p (Figure 7(c), *P* = 0.0279, *R*^2^ = 0.146), consistent with the fact that A*β* and APP represent two consolidated miR-195-5p targets [62].

A*β* accumulation was not significantly related to the other examined parameters (BACE1: *P* = 0.0957; HDAC6: *P* = 0.3744; and miR-153-3p: *P* = 0.1787).

Of note, erythrocyte BACE1 levels showed an inverse correlation with plasma miR-153-3p (Figure 7(d), *P* = 0.0415, *R*^2^ = 0.211) [49].

## 4. Discussion

**Figure 8:**
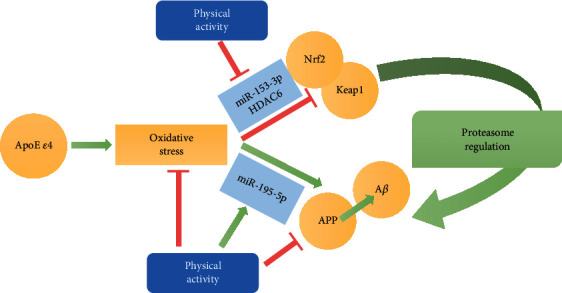
Influence of ApoE *ε*4 polymorphism and physical activity in the pathways of the proteasome system. The presence of ApoE *ε*4 polymorphism was associated with an elevated concentration of the proteasome-related Keap1, which inhibited Nfr2 and impaired the proteasome system. These events lead to an enhancement of A*β* and miR-153-3p, as well as to minor plasma AOC. Aside from ApoE polymorphism, regular physical exercise reduced oxidative stress, by increasing the plasma antioxidant capability, and blocked the pathway of A*β* production/accumulation. Physical exercise can also regulate the epigenetic mechanisms involved in the Nrf2-Keap1 axis and A*β* production by reducing miR-153-3p and HDAC6 and enhancing miR-195-5p expression.

The current study evaluated the impact of ApoE polymorphism and oxidative stress on the amyloidogenic pathway in peripheral blood cells; moreover, the protective effects of physical activity in reactivating the proteasome system were explored. The main results of the paper are as follows (Figure 8): (i) plasma AOC was significantly reduced in ApoE *ε*4 subjects; (ii) the concentration of A*β*, the proteasome-related Keap1, HDAC6, and the Nrf2 blocker, miR-153-3p, was augmented in *ε*4 carriers compared to non-*ε*4 carriers; (iii) physical exercise was associated with increased plasma AOC and a reduced amount of A*β* and of its precursor APP; and (iv) physical exercise-induced effects involved a reduced expression of Keap1, HDAC6, and the Nrf2 blocker, miR-153-3p, together with an enhancement of miR-195-5p. Taken together, our study highlights the impact of the ApoE genotype on the amyloidogenic pathway and the proteasome system and suggests the positive impact of physical exercise on the protective mechanisms against A*β* accumulation and proteasome inhibition, also through the modulation exerted by epigenetic mechanisms.

However, additional statistical analysis by two-way repeated measures ANOVA suggested that, while physical activity was not a significant factor, significant differences were observed in discrepancy among genotypes (*P* < 0.05).

ApoE polymorphism plays a pivotal role in the transport and metabolism of lipids. Moreover, it has been widely implicated in the modulation of the oxidative status and misfolded proteins' accumulation, even before the onset of neurological diseases [6]. On the other hand, regular physical activity has been proven to reduce the accumulation of toxic oligomers and modulate the levels of oxidative stress, finally enhancing neurogenesis and counteracting neurodegeneration processes [42, 63].

Herein, erythrocytes and plasma were elected as a good peripheral model to investigate neurodegeneration-related proteins, because they are particularly susceptible to oxidative stress and capable of accumulating misfolded proteins [46, 47, 64]. By taking advantage of this peripheral model, the present study was aimed at investigating the influence of the ApoE polymorphism and physical activity on the oxidative stress levels, the amyloidogenic pathway of A*β* production, and the Nrf2-mediated proteasome functionality.

Oxidative stress has been proven to increase in ApoE *ε*4 carriers and to rise with aging [42, 65]. Accordingly, plasma AOC, an indirect measure of oxidative stress, was significantly lower in the presence of *ε*4 polymorphism. Nevertheless, plasma AOC was independent of age, probably because of the poor age interval of the enrolled subjects. Furthermore, physical exercise enhanced the antioxidant capability, independently of ApoE polymorphism, thus confirming previous reports [42, 45]. In particular, plasma AOC showed a significant positive correlation with the level of physical activity, as previously described in human subjects [42, 43, 45, 66].

As noticed for plasma oxidative status, A*β* accumulation in erythrocytes occurred particularly in ApoE *ε*4 carriers, confirming that this polymorphism plays a pivotal role in A*β* deposition [5, 67], even in peripheral fluids [68]. Although A*β* originates from its precursor APP, no significant differences were evidenced between ApoE *ε*4 carriers and noncarriers. These results suggest that the polymorphism does not influence the initial amount of APP but rather its processing through the amyloidogenic pathway. Nevertheless, our results showed an inverse correlation between APP and A*β* with plasma AOC towards hydroxyl radicals, confirming the link between oxidative stress and the amyloidogenic pathway.

A*β* is generated from APP through the rate-limiting enzyme BACE1. Increased BACE1 levels and activity have been reported in the brain of patients with sporadic AD and well correlated with its end product A*β* [69]. Surprisingly, in our hands, BACE1 concentrations were significantly higher in non-*ε*4 carriers than in *ε*4 carriers. Additional experiments will be required to measure the enzyme activity, rather than its concentration, to verify putative differences among the different ApoE polymorphisms. In this respect, BACE1 activity in platelets has been found to be increased in AD patients [70]. Of note, the use of different blood cells (i.e., platelets versus erythrocytes) may be another cause explaining our data. Finally, an additional explanation may come from putative BACE1 polymorphisms carried by the subjects [71].

As concerns the impact of physical exercise on the amyloidogenic pathway, APP and A*β* levels in erythrocytes were significantly lower in active subjects compared to non-active ones, thus confirming that physical exercise modulates A*β* production and accumulation. Consistent with the data discussed above, BACE1 decreased with physical activity in the absence of ApoE polymorphism. In particular, APP and A*β* concentrations strictly and inversely depended on the level of physical exercise in the whole group. These data are consistent with those reporting that physical exercise can reduce A*β* synthesis and accumulation in plasma [72] and erythrocytes [42, 43, 45].

Accumulating evidence indicates that neuroinflammatory and neurodegenerative processes are triggered by the dysfunction of the UPS [11, 73]. In particular, decreased levels and transcriptional activity of the nuclear factor Nrf2 contribute to the propagation of oxidative stress and neurodegeneration-related proteins [12]. Considering the strict link between the proteasome system and misfolded protein accumulation, we investigated the impact of ApoE polymorphism and physical activity on the modulation of the UPS pivotal actors.

Herein, the erythrocyte concentrations of Nrf2 did not change between ApoE non-*ε*4 carriers and *ε*4 carriers. In contrast, Keap1 levels decreased in non-*ε*4 carriers compared to *ε*4 carriers. Accordingly, Keap1 has been proven to inhibit Nrf2, and the direct inhibition of Keap1 has demonstrated to reactivate Nrf2 in neurodegeneration [74]. Consistent with the Keap1 inhibitory role on Nrf2, Keap1 concentration was significantly lower in active subjects in the *ε*4 carrier subgroup. According to our data, exercise trials have been proven to significantly increase the nuclear Nrf2 levels in different peripheral tissues [75, 76].

In order to investigate the epigenetic mechanisms around the Keap1-Nrf2 axis [77], the expression of the HDAC6 and two specific miRNAs was investigated. Indeed, HDAC6 has been proven to inhibit the transcription factor Nrf2, and HDAC inhibition can reduce Keap1-mediated Nrf2 suppression, Nrf2 nuclear translocation, and Nrf2 binding to antioxidant response elements [78]. In our hands, HDAC6 concentration in erythrocytes was lower in non-*ε*4 carriers than in *ε*4 carriers, consistent with the trend obtained for Keap1 in the same subjects.

Finally, the expression of miR-153-3p and miR-195-5p was explored. Among the different miRNAs nowadays implicated in neurodegeneration, miR-153-3p was chosen because of its link with the Keap1-Nrf2 axis and the downstream genes [79, 80], while miR-195-5p was chosen for its ability to downregulate the transcriptional expression levels of BACE1 and APP [26, 62]. The negative correlation observed in this study between both A*β* and APP concentrations and miR-195-5p is consistent with strong modulation of A*β* and APP by miR-195-5p [62]. Furthermore, miR-153-3p, whose expression was significantly lower in subjects not carrying *ε*4, inversely correlated with BACE1 levels. Accordingly, miR-153-3p has been proven, in animal models and AD patients, to target directly APP by binding to its 3′ UTR, finally downregulating A*β* [24, 81].

Interestingly, miR-153-3p also showed a negative correlation with plasma AOC towards hydroxyl radicals. In contrast, miR-195-5p was positively related to plasma AOC. The same miRNA-related correlations were observed with the level of physical exercise, suggesting that the two miRNAs positively and negatively regulate the redox response to physical exercise [49].

Therefore, these data provide a deeper understanding of the molecular mechanisms underlying the beneficial effects of physical exercise that, with other environmental and lifestyle factors, may influence epigenetics and miRNA expression [38, 39].

## 5. Conclusions

In conclusion, in the present paper, we showed that ApoE *ε*4 polymorphism was associated with an elevated concentration of A*β*, the proteasome-related Keap1, and miR-153-3p, as well as with minor plasma AOC. Independently of ApoE polymorphism, physical exercise was associated with increased plasma AOC and reduced the amount of A*β* and of its precursor APP. Moreover, physical exercise-induced effects involved a reduced expression of Keap1, HDAC6, and miR-153-3p, together with an enhancement of miR-195-5p.

Overall, our study highlights the impact of the ApoE genotype on the amyloidogenic pathway and the proteasome system and evidenced the positive effects of physical activity against A*β* accumulation and proteasome inhibition, also through epigenetic mechanisms that involved miR-153 and miR-195-5p (Figure 8) [49].

Remarkably, the obtained results shed light on the impact of ApoE polymorphism on the molecular mechanisms involved in the regulation of A*β* accumulation and the Nrf2-Keap1 pathway in peripheral cells, focusing on their modulation by the physical activity and epigenetic mechanism, like HDAC6 and miRNA involvement.

However, these data are only potentially reliable and should be further investigated taking also in consideration other environmental parameters, such as firstly the diet, with particular regard to vitamin D, calcium, and selenium, which could modulate the investigated pathways, i.e., the amyloid production, antioxidant defence, and proteasome activity, concerning the ApoE polymorphism.

Nevertheless, the current study highlights putative peripheral biomarkers for AD development and candidate targets that could be modulated by lifestyle, such as physical activity.

The novelty of our study lies in the investigation of altered pathways in relation to ApoE polymorphism, which usually occurs in the CNS and in peripheral cells, and the modulation of these is due to physical activity. Therefore, the positive impact of physical activity, even in the presence of ApoE *ε*4 polymorphism, could represent a pivotal tool to investigate novel peripheral targets for putative preventive treatments for AD.

Finally, the use of blood, a fluid that can be easily and cheaply collected without invasive procedures, consolidates the study. Particularly, the use of erythrocytes as a good model to investigate the biochemical alterations that occur in AD, and more generally in NDs, allows easily exploring molecular alterations, which arise many years before the onset of clinical symptoms, and their modulation.

## Figures and Tables

**Table 1:** Cohort of healthy volunteers who engaged in the study.

Groups	Number of subjects (*N*)	Age (years)	Sex (M/F)	Physical activity level (Borg scale)
ApoE *ε*4 carriers	16	39 ± 14	7/9	9 ± 3
ApoE non-*ε*4 carriers	26	40 ± 13	12/14	10 ± 4
NA ApoE *ε*4 carriers	8	38 ± 11	3/5	7 ± 1
A ApoE *ε*4 carriers	8	41 ± 21	4/4	13 ± 2
NA ApoE non-*ε*4 carriers	13	41 ± 14	5/8	7 ± 1
A ApoE non-*ε*4 carriers	13	39 ± 12	7/6	13 ± 2

## Data Availability

The data used to support the present findings are available from the corresponding author upon request.

## Abbreviations

BACE1: *β*-Secretase 1
AD: Alzheimer's disease
A*β*: Amyloid *β*
APP: Amyloid precursor protein
ARE: Antioxidant response element
ApoE: Apolipoprotein E
CNS: Central nervous system
HDAC6: Histone deacetylase 6
Keap1: Kelch-like ECH-associated protein 1
NDs: Neurological disorders
Nrf2: Nuclear factor erythroid 2-related factor 2
ROS: Reactive oxygen species
AOC: Total antioxidant capability
TOSC: Total oxyradical scavenging capacity
UPS: Ubiquitin-proteasome system.
